# A New Trend in Recording Subgingival Tissue around an Implant While Making a Direct Abutment Impression

**DOI:** 10.1155/2014/847408

**Published:** 2014-06-01

**Authors:** Suryakant C. Deogade, Sneha S. Mantri, Gunjan Dube, Radhika Shrivastava, Syed Noorani

**Affiliations:** ^1^Department of Prosthodontics, Hitkarini Dental College & Hospital, Jabalpur, Madhya Pradesh 482005, India; ^2^Department of Oral Surgery, Hitkarini Dental College & Hospital, Jabalpur, Madhya Pradesh 482005, India

## Abstract

A successful implant-supported restoration must provide adequate function and esthetics. Osseointegrated implants have given an alternative choice for patients who have lost their teeth. Most commonly encountered problems while doing a transfer from patient to the master cast in restoring implant-supported crowns are an uneven distribution of occlusal loads and undue torquing forces on the various elements of implant. This is caused due to poor fit of frameworks connected to implant, which further leads to marginal bone loss, loosening of screws, fatigue fracture of implant components, and ultimately implant failure. This paper presents a simplified and easy solution to overcome such problems by introducing an innovative gingival retraction system for restoring implant-supported crowns to achieve superior and predictable long-term outcomes.

## 1. Introduction

Osseointegrated implants have proven predictable long-term alternative treatment options to traditional prostheses for patients who lost their teeth [1, 2]. For predictable long-term benefits, an accurate and passively fitting prosthesis and successful surgical procedure have been recommended as the prime and critical requirements [3–8]. The traditional transfer impression techniques seldom deliver a passive fit of a framework which ultimately causes a failure of most of the implant-supported prosthesis. Several studies showed that transfer technique is almost four times worse than the clinical requirement.

The poor fit of frameworks connected to implant may cause uneven distribution of occlusal loads and torquing stresses. These problems may lead to marginal bone loss, loosening of screws, fatigue fractures of implant components, and failure of implants [4–10]. The precise transfer of the spatial relationships of implants from the oral cavity to the master cast with an impression is the first and critical step to ensure passive fit of implant framework. Therefore, improving the transfer accuracy of the impression copings becomes an utmost important task in the clinical practice [11–13].

However, the inaccuracy in dental implant impression is a major and unsolved problem. This can drastically affect the osseointegration of the majority of implants. This case report describes a solution for such problems, by using a newly introduced gingival retraction system for making a direct abutment-level impression in routine clinical practice.

## 2. Case Report

A 37-year old female patient reported to the department of prosthodontics with a request to discuss the options for prosthetic replacement of mandibular right first molar which was extracted 6 months back. She was given an option of implant-supported crown. The patient agreed to proceed with an implant-supported crown with 36. Clinical and radiological assessment showed an adequate bone support with 36. The benefits and the risks of the planned treatment were explained to the patient and a signature for consent was obtained.

Recently, a Canadian company, Stomatotech, came up with a simple idea to retract the gingival tissue using a disposable plastic collar that is inserted on the apical end of the abutment before the abutment is engaged to the implant. Following the abutment's engagement to the implant, the plastic collar is found between the apical part of the abutment and the gingival soft tissue. Shortly after the removal of the impression from the mouth, the plastic collar is pulled out and removed permanently. The plastic collar creates a perfect gingival retraction with a valve factor preventing the liquids from contaminating the area of the finish line of the abutment.

**Figure 1:**
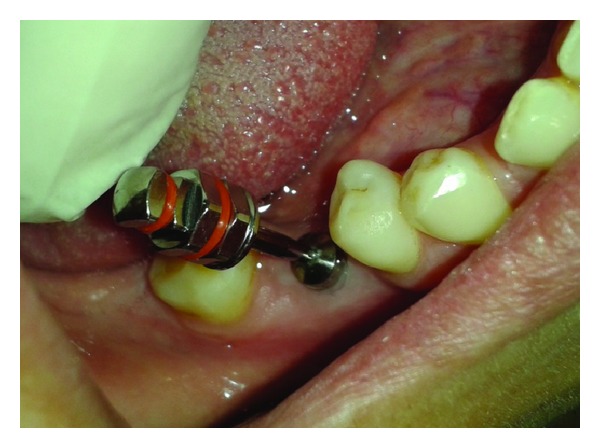
Healing abutment removed.

**Figure 2:**
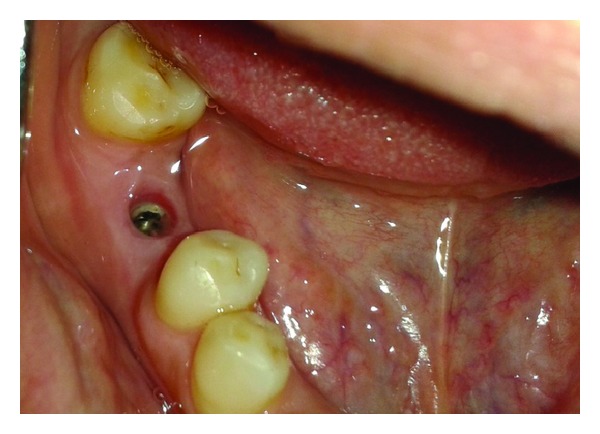
Implant with surrounding tissue.

**Figure 3:**
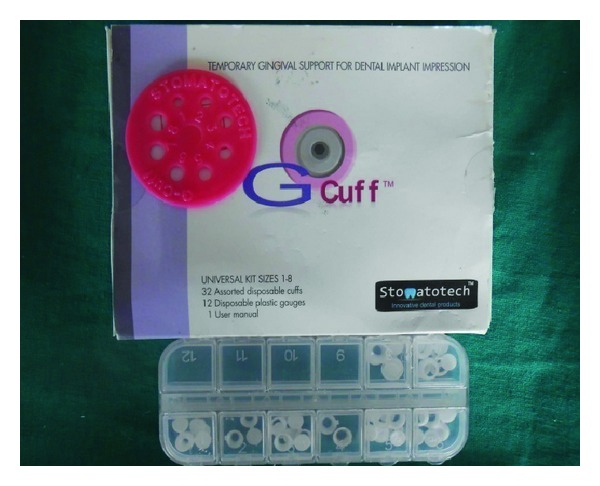
G-Cuff kit.

**Figure 4:**
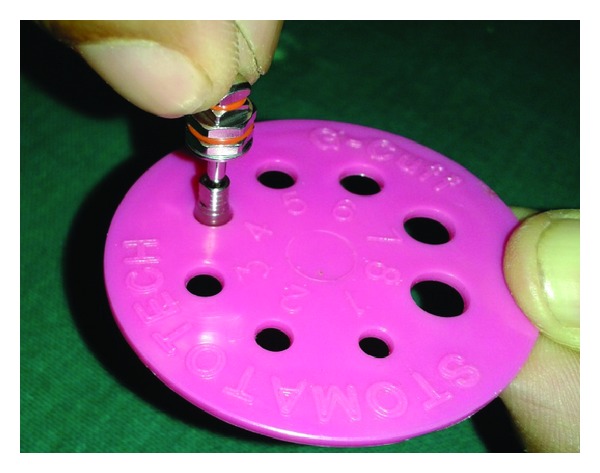
Selection of G-Cuff.

**Figure 5:**
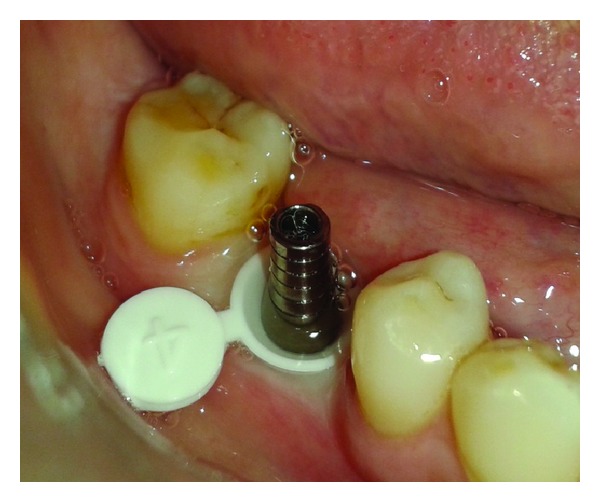
Abutment along with G-cuff.

**Figure 6:**
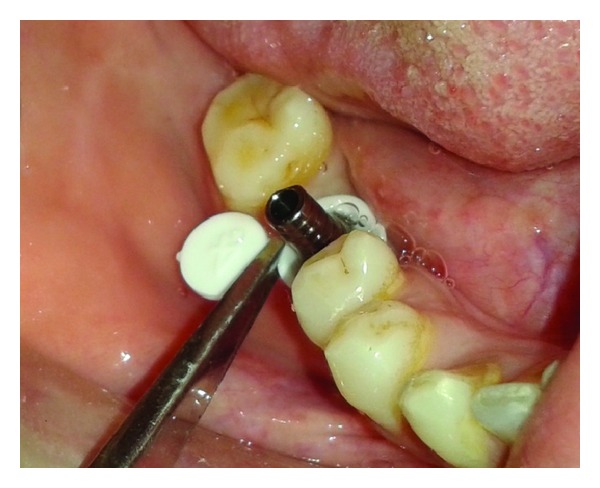
Extraportion cut with scissor.

**Figure 7:**
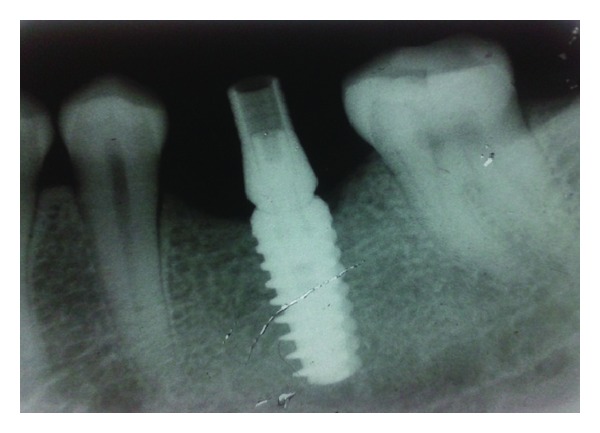
IOPA of implant-abutment assembly.

**Figure 8:**
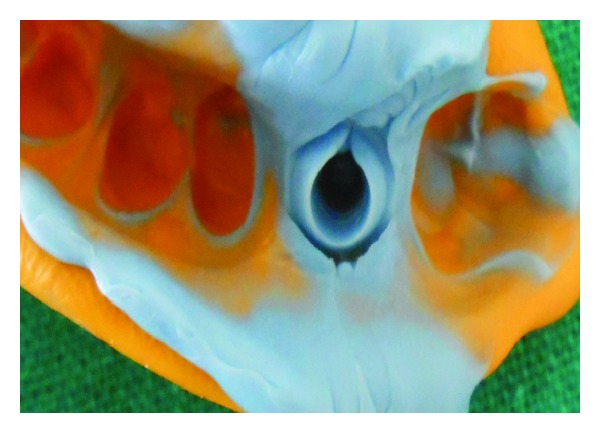
PVS impression.

**Figure 9:**
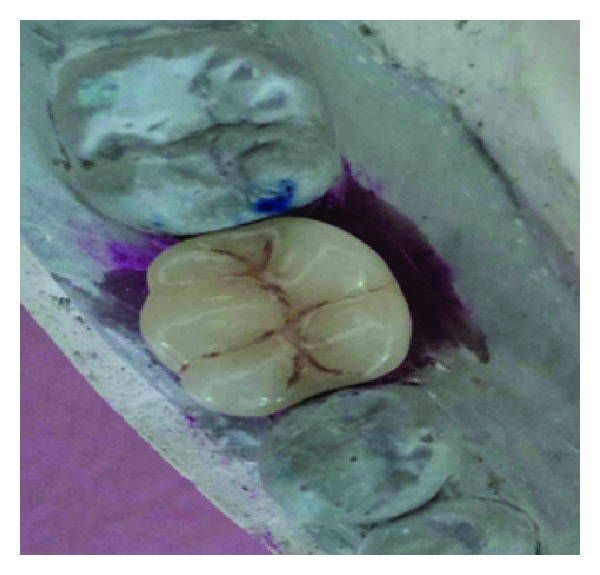
Definitive crown.

**Figure 10:**
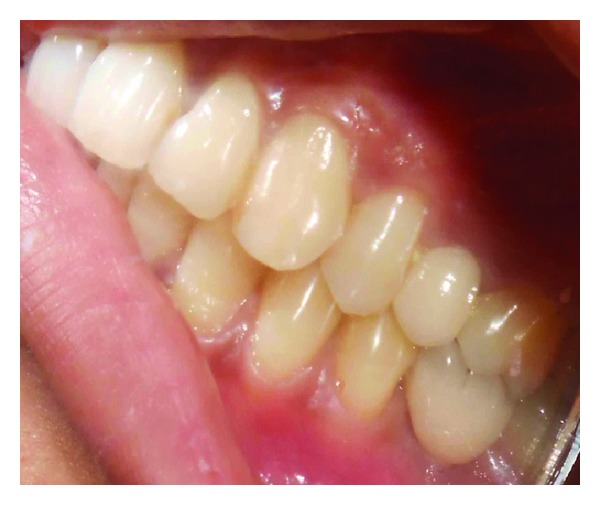
Cementation of crown.

**Figure 11:**
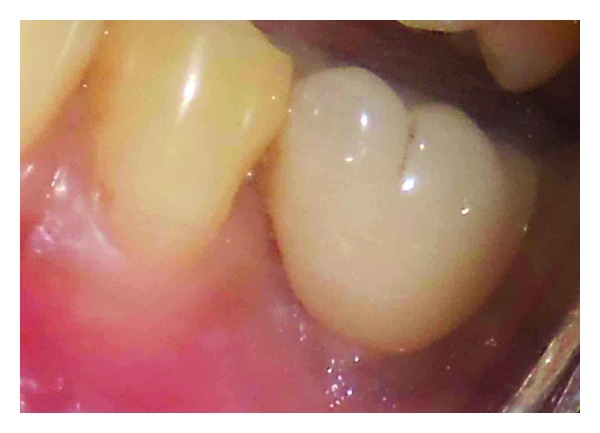
Emergence profile of crown.

**Figure 12:**
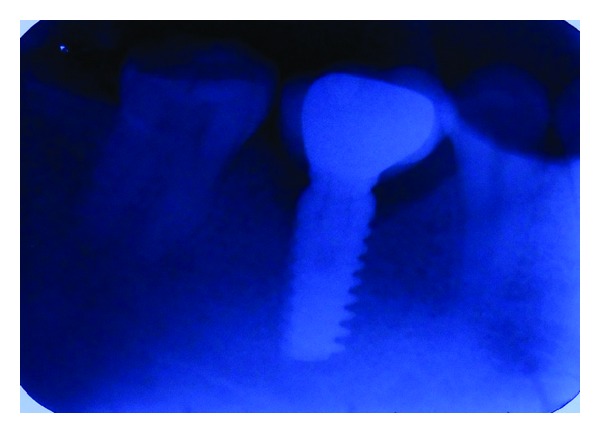
IOPA after one-year.

**Figure 13:**
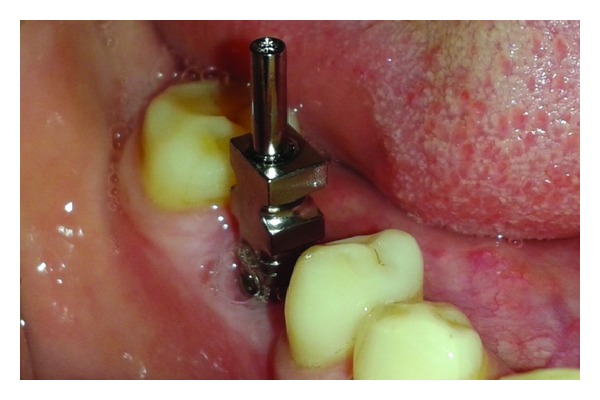
Transfer coping attached.

**Figure 14:**
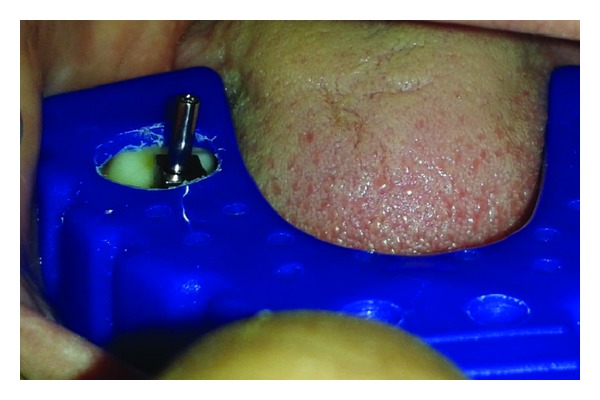
Window prepared in the tray.

**Figure 15:**
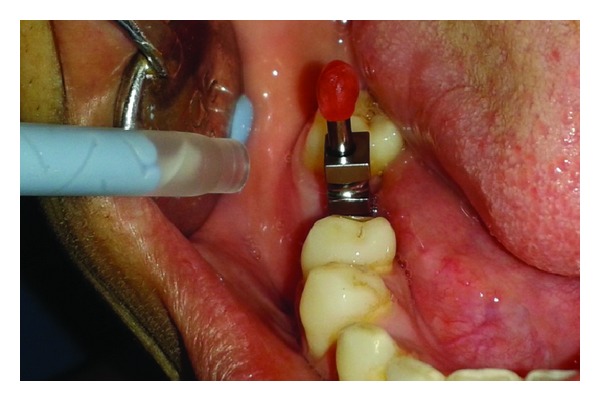
Coping screw blocked with wax.

**Figure 16:**
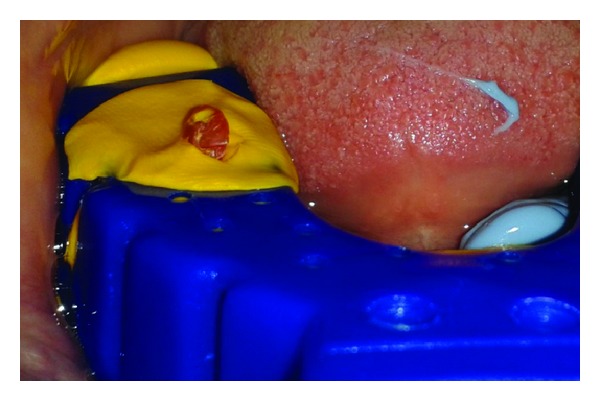
Coping screw exposed.

**Figure 17:**
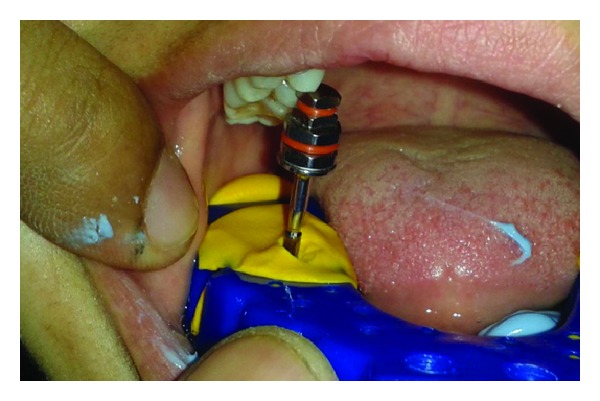
Coping screw removed.

**Figure 18:**
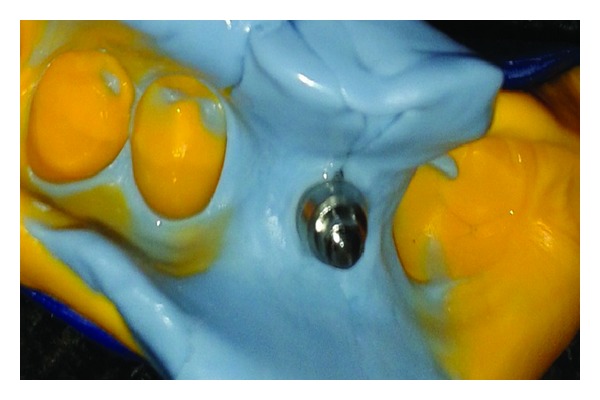
PVS impression without G-Cuff.

The patient was called after three months for prosthodontic intervention. The healing abutment was removed (Figure 1) and the gingival tissue surrounding the implant was assessed clinically (Figure 2). The G-Cuff (Stomatotech, Canada) (Figure 3) was planned to temporarily support the gingival surrounding the implant before making a direct-abutment level impression. The prepared abutment was checked in the special G-cuff measuring tool (Figure 4). After determining the size of the cuff, the correct one G-Cuff collar was picked from the kit. The G-Cuff was placed, and the abutment was screwed back to the implant fixture (Figure 5). The screw driver was fastened to the torque of 20 Ncm. The extra part of the collar was cut (Figure 6) and a periapical radiograph was obtained (Figure 7). Then the direct PVS impression was taken (Figure 8) and sent to the lab. A metal-ceramic crown was fabricated (Figure 9). The definitive crown was placed, and only minor occlusal adjustments were needed (Figures 10 and 11). The patient was called for regular check-up and a periapical radiograph was obtained after six months (Figure 12). To show the comparison between impressions with G-Cuff and without G-Cuff, one more impression was made with open tray technique. A square impression coping was screwed to the implant fixture (Figure 13) and the top of screw was blocked with wax (Figure 15). After that, a suitable stock plastic tray was selected and a window was created over the region of coping (Figure 14). Then a PVS impression was obtained as described in open tray technique (Figures 16, 17, and 18). The comparison between both impressions shows that G-Cuff records peri-implant tissues in a similar way as that of conventional method with gingival retraction.

## 3. Discussion

The utmost care is needed while doing implant transfer to obtain master casts and also while achieving a passive fit of the prosthetic superstructure to the implant to maintain the osseointegration around the implant [14–19]. Eames et al. [20] suggested the importance of impression procedures to obtain suitable and reliable prosthetic superstructures, which can be achieved with the following steps: impression, cast acquisition, waxing, embedding, and casting. Numerous techniques of implant transfer are reported, but its accuracy is still questionable [18, 21]. Recently, the main impression techniques practiced are closed tray with tapered copings and open tray with square copings, which may be used together or not [22–24].

The problems encountered while doing the transfer can be enlisted as follows.

1. The transfer coping while impressing is mechanically caught in the impression material. It does not become an integral part of the impression and can be easily moved.
2. Also, when the lab technician engages analogs into the impression, its displacement cannot be avoided.
3. The displacement of the transfer coping can also happen due to the gravity forces of the impression tray (especially in the molar areas). An impression tray that weighs 100 grams can generate a torque of 5.8 Ncm by its own weight, which is sufficient to shift the transfer.
4. Splinting with acrylic resins may lead to displacement of the transfers due to the shrinkage of the acrylic materials. Even a splinted assembly of impression transfers does not become an integral part of the impression.
5. Due to the expansion of the dental stone during its setting, a serious shifting of the analog from its original position is caused. Hence, the analog becomes loose and mobile.
6. The sectioning of an implant stone model is very difficult because of the presence of the hard steel analogs in the body of the model. Also, a small amount of the stone around the analogs often leads to breakage of the die. This needs either a redo of the master cast or working on an unsectioned cast. All these difficulties prevent precise fabrication of the implant-supported restoration.

The prime concern with the direct impression is the abutment's subgingival area registration. Dr. Vincent Bennani [25] discussed the gingival retraction techniques for implants versus teeth. He covered most gingival retraction means for natural teeth and concluded that there is no existing device or method for gingival retraction that can be practically used for direct impression of the implant abutment.

Chang et al. [26] studied the effects of a cordless retraction paste material on implant surfaces. They observed minimal changes in the implant surface morphology and composition after Expasyl contact. Bicon Implants use oversized healing abutments or custom oversized temporary abutments to expand the surrounding tissue. This method has little predictability because the rebound of the tissue varies from patient to patient.

The main purpose of G-Cuff is to support soft tissue that surrounds the implant abutment allowing the impression means (conventional or digital) to have an access to the surface of the abutment needed for the optimal restoration. The restoration with G-Cuff is a two-appointment procedure which completely eliminates a “try-in” visit. It renders a simple, affordable, and universal solution for any bridge and crown restoration.

The advantages of this simple and efficient system are both economical and clinical as follows.

1. As accuracy is the main factor in a restoration procedure especially for bridges or splinted crowns, the impression with G-Cuff is way more reliable than any other method, either “open tray” or “close tray.” Due to the significant shortening of the lab procedure, the risk of impression distortion is dramatically lowered.
2. The G-Cuff system eliminates the need of materials such as impression copings, implant analogs, temporary abutments, and custom impression trays.
3. In addition, the system solves a problem of the U.D.I (unidentified dental implants). The original abutment and a G-Cuff are enough to take a new impression and then to complete the restoration without even knowing the brand of the implant.
4. The list of the advantages also includes reduction of redo rate, universality, chair-time reduction, precision of a framework, and the fact it works with custom/stock abutments.
5. It also acts as a cement barrier making the peri-implant area free of cement remains. G-Cuff keeps the occlusal part of the gingival margin open, letting cement flow outside, as well as seals the apical part of the gingival margin, thus preventing cement from penetrating the Peri-implant tissue.
6. It maximizes infection control due to the minimal manipulation of the abutment, and also numbers are printed on each G-cuff (8 different sizes) for easy recognition.
7. Unlike the retraction cord, G-Cuff does not traumatize the tissue around the implant.
8. It allows sectioning of any stone model creating a perfect working condition for the lab. In many cases transfers sectioning of the stone model is just impossible or very hard. G-Cuff eliminates any metal parts (an analog) in the stone model allowing an easy sectioning like for regular crowns and bridges.
9. Due to a special design of the apical margin of the cuff when the abutment is tightened, the cuff stretches and slides out of the AIJ (abutment implant junction) and is never trapped.
10. In the cases where the aesthetic demand is extremely high, only G-Cuff allows the technician to access the entire restoration to create the utmost aesthetics.

## 4. Conclusion

The problems while making transfer of implant impression from patient's mouth to the definitive cast are numerous, which cause poor fit of framework and finally implant failure. A slight shift or rotation of transfer is unavoidable causing an obstacle in the success of implant-supported restorations. A newly introduced gingival retraction system (G-Cuff) was used in the described case report which yielded a successful and predictable restoration for the patient. This retraction system can be advocated to single crowns and bridges for the long-term outcomes.

The G-Cuff kit is a must in every dental office because it is the only solution for UDI (unidentified dental implants), which recently have become more and more frequent.
